# Remote assessment of quality, processing and economic criteria in qualification of suppliers to the meat packaging foil producer—A Greek case study

**DOI:** 10.1371/journal.pone.0278021

**Published:** 2023-02-06

**Authors:** Katarzyna Jakubowska-Gawlik, Wojciech Kolanowski, Dimitris Mantzoros, Ewa Czarniecka-Skubina, Joanna Trafialek

**Affiliations:** 1 Institute of Human Nutrition Sciences, Warsaw University of Life Sciences (WULS), Warsaw, Poland; 2 Faculty of Health Sciences, Medical University of Lublin, Lublin, Poland; 3 Flexopack S.A, Koropi, Attiki, Greece; Universita degli Studi del Molise, ITALY

## Abstract

Supervision over the suppliers of packaging as well as suppliers of raw materials for packaging production is important to ensure the quality and safety of meat products. The aim of this study was to verify the remote evaluation procedure of quality, processing and economic criteria in qualification of raw materials suppliers to the meat packaging foil producer during the Covid-19 pandemic. The evaluation was done remotely in terms of meeting some of the requirements of the quality management system (QMS) in conditions where regular audit could not be carried out. The survey was conducted in one of the biggest packaging foil producers in Greece via its supplier evaluation. The evaluation consisted of: 1/ economic criteria and 2/ quality and processing criteria. The highest and the lowest rated economic criteria were procedural compliance and price of raw materials. Among the quality and processing criteria, the highest score was given to warranties and claims policies and material quality, and the lowest one to lead time. The highest ratings obtained suppliers of raw materials directly involved in production, suppliers from Greece, the USA and Denmark, as well as suppliers to the R&D department. The results of the study showed that the quality of the raw materials directly used in the production of packaging foil was adequate. Therefore, their use ensure production of packaging foil and finally packaged meat products of adequate quality and safety. The presented procedure occurred to be useful for remote evaluation of quality, processing and economic criteria in qualification of suppliers during the Covid-19 pandemic. It may inspire other producers of food packaging materials to continuing supervision over their suppliers while regular methods of control are limited.

## Introduction

Supervision over the suppliers of food packaging as well as suppliers of raw materials for packaging production is important to ensure the quality and safety of meat products. The quality of the final meat product is determined by many factors. The most important are the production process, the raw materials quality, packaging and storage conditions [1, 2]. The quality management system (QMS) helps meet regulatory standards and requirements, which are necessary to achieve the good quality of food products. The QMS documents the policies, business processes and procedures for achieving quality control objectives. The primary goal of the QMS is compliance with regulatory requirements [3, 4]. In the case of raw materials, the selection of suppliers and their regular evaluation are the key factors of quality management [5–12]. Therefore, enterprises which have implemented and certified food safety systems and a QMS should implement continuous evaluation of suppliers [3, 13–15]. For this reason, each organisation should determine the supplier evaluation criteria to guarantee the required quality of the raw materials and ingredients which are to be supplied and used in production [3, 16]. The common set of supplier selection criteria includes price, delivery, quality, and service is considered universal for most industries [17]. Moreover, other important criteria are supplied materials quality [18, 19], lead time [19, 20], product reliability [20], packaging capabilities [21], the technical capabilities of the supplier [22] (Ho et al., 2010), honest and frequent communication [22, 23], relationships [23], and ethics [19, 20, 24]. In the case of fresh food raw materials, cold chain is another important criterion [25]. It is also required to take into account social criteria like equality, gender non-discrimination, working hours, no child labour, decent earnings and additional benefits [26, 27]. Sometimes, it is important to extend the evaluation to sustainable approaches, such as the use of green or recyclable packaging, in order to reduce environmental pollution and climate protection [6, 24, 28].

Nowadays, food producers often lack procedures for evaluating their suppliers [29]. Usually, it is done by third-party audits. However, one-off audits of suppliers are not reliable enough as some nonconformities may not be identified during the audit. Moreover, conducting regular audit is not always possible, like during the Covid-19 pandemic. Therefore, in terms of QMS, it is necessary to elaborate and adopt remote methods of supplier evaluation, which may be carried out instead of regular audit.

In general, there are not many publications dealing with the evaluation of suppliers for the food industry manufacturers. In a few studies, suppliers were assessed on the risk of cooperation [16, 30–32]. The evaluation criteria concerned supplier selection and supervision [10, 33–37]. However, there are no papers presenting the evaluation of suppliers for producers of food packaging foil for meat products in terms of food safety requirements and QMS [3]. Moreover, there is also lack of reports describing supplier evaluation procedures in special conditions like the Covid-19 pandemic, when external regular audits are limited or cannot be carried out at all. This study fills this gap by developing, presenting and testing a remote procedure of some QMS criteria of suppliers evaluation, based on real case of the foil producer.

Nowadays, food producers often lack procedures for evaluating and choosing their suppliers [29]. Usually, they use third-party audits and rely on their results. However, one-off audits of suppliers are not reliable enough as some nonconformities may not be identified during the audit. Moreover, conducting regular audit is not always possible, like during the Covid-19 pandemic. Therefore, in terms of QMS, it is important to adopt remote methods of supplier evaluation, which may be carried out without regular audit. The evaluation of suppliers is not single-criterion decision-making issue. There are other quantitative and qualitative factors that should be considered [34]. The aim of this study was to verify remote evaluation procedure of quality, processing and economic criteria in qualification of raw materials suppliers to the meat packaging foil producer during the Covid-19 pandemic. The evaluation was done remotely in terms of meeting some of the requirements of the QMS in conditions where regular audit could not be carried out.

## Methods

### Procedure of actions

The evaluation was based on requirements of ISO 9001 point 8.4 (Control of externally provided processes, products and services) [3]. The survey was conducted remotely among suppliers of the large-scale producer of foil intended for food packaging, especially in the meat industry (Flexopack, Greece). The producer supplies packaging foils to meat processing plants in many countries. The time of the survey was the year 2020 during the Covid-19 pandemic, when the regular audits by external auditors could not be carried out. The procedure of actions included: 1/ review of the literature, contracts with suppliers and the results of previous suppliers audits; 2/ preparation of the questionnaire (selection of criteria for the evaluation of supplies and the scoring scale); 3/ determination of the potential use of the developed procedure in the suppliers qualification; 4/ conducting the research; 5/ statistical multi variant analysis of the obtained results.

### Questionnaire

The three-part evaluation questionnaire consisted of two evaluation sections concerning, 1) economic criteria, 2) quality and processing criteria and additional section concerning the information about the supplier. The questionnaire was developed by the authors of this study based on previous studies reporting on supplier selection and evaluation [6, 35, 38, 39]. The economic part contained six subcriteria, which were: price, financial position, desire for business, procedural compliance, cooperation and business ethics (Table 1). Meanwhile, the quality and processing section included seven subcriteria: quality of raw materials, lead time, quality certifications [3, 14, 29], production facilities and capacity, technical capability, service/technical assistance, warranties and claims policies.

**Table 1:** The scope of the supplier assessment and supplier information.

No.	Criteria	Score	Definition
1. **Economic criteria**			
1.1	Price	0 –very high	Price higher than average market prices by more than 15%, non-negotiable
		1 –high	Price higher than average market prices by 15%, negotiations possible
		2 –medium	Average market price comparable to the competition
		3 –low	Price lower than market prices by 15%
1.2	Financial position	0 –very low	Market reports showed losses and problems with the company’s financial liquidity
		1 –low	No reported profits in market reports, very short payment terms
		2 –medium	Market reports showed low profits, short payment terms
		3 –high	Market reports showed profits, long payment terms
1.3	Desire for business	0 –very low	Very low desire–supplier irrelevant to the producer, it is possible to withdraw from cooperation without affecting the producer’s activity
		1 –low	Low desire–neutral supplier, withdrawal from cooperation may have a slight negative impact on the producer’s activity
		2 –medium	Desired supplier–withdrawal from cooperation may have a large negative impact on the producer’s activity
		3 –high	The most desirable supplier–withdrawal from cooperation may pose a threat to the producer’s activity
1.4	Procedural compliance	0 –very low	The supplier does not respect the cooperation procedures
		1 –low	The supplier respects some of the cooperation procedures
		2 –medium	The supplier respects most of the cooperation procedures
		3 –high	The supplier respects all agreed cooperation procedures
1.5	Cooperation	0 –very low	Significant difficulties in receiving responses to correspondence
		1 –low	Difficulties in receiving responses to correspondence
		2 –medium	Seamless exchange of correspondence
		3 –high	Very seamless exchange of correspondence
1.6	Business ethics	0 –very low	The supplier does not adhere to ethical standards
		1 –low	The supplier adheres to some ethical standards
		2 –medium	The supplier adheres to most ethical standards
		3 –high	The supplier adheres to all ethical standards
**2.**	**Quality and processing criteria**		
2.1	Material quality	0 –very low	Quality below accepted specifications
		1 –low quality	Nonconformities occur with each delivery
		2 –acceptable quality	Nonconformities occur every 2–5 deliveries
		3 –high quality	No nonconformities in the last year
2.2	Lead time	0 –very low	Delivery over 4 days
		1 –long lead time	Delivery within 4 days
		2 –average lead time	Delivery within 3 days
		3 –short lead time	Delivery within 2 days
2.3	Certification	0 –not acceptable	Loss of certification by supplier
		1 –acceptable	One certification
		2 –good	Two certifications
		3 –excellent	Three or more certifications
2.4	Production facilities and capacity	0 –very low	No high-volume production capacity, no other plant locations
		1 –low	Low production capacity; ability to produce large quantities in a short time with great constraints, other plant locations available
		2 –medium	High production capacity; ability to produce large quantities in a short time with some restrictions, other plant locations available
		3 –high	Very high production capacity; ability to produce large quantities in a short time, other production locations available
2.5	Technical capability	0 –very low	Outdated supplier production technologies, no R&D department
		1 –low	Low level of advanced supplier production technologies, poorly developed R&D department
		2 –medium	Medium level of advanced supplier production technologies, developed R&D department
		3 –high	Very advanced supplier production technologies, highly developed R&D department
2.6	Service/Technical assistance	0 –very low	Service and technical support available within 3 days or more
		1 –low	Service and technical support available within 2 days
		2 –medium	Service and technical support available within 1 day
		3 –high	Service and technical support available within 4 hours
2.7	Warranties and claims policies	0 –very low	Lack of cooperation in considering complaints
		1 –low	Long wait time for complaints handling and responses to complaints
		2 –medium	Short wait time for complaints handling and responses to complaints
		3 –high	Very short wait time for complaints handling and responses to complaints
3	**Supplier information (not scored—ns)**		
3.1	Supplied materials	ns*	Raw materials directly involved in production
		ns	Raw materials indirectly involved in production
3.2	Geographical location (Country)	ns	Greece
		ns	USA
		ns	Denmark
		ns	Spain
		ns	Germany
		ns	France
		ns	Italy
		ns	UK
3.3	Department	ns	Raw materials for production department
		ns	Raw materials for research and development department
		ns	Raw materials for logistics department

*ns–not scored

A grading scale was used for the evaluation of each question: the highest score was 3, which corresponded to all expectations being met (i.e. 90–100% compliance with requirements); a score of 2 reflected a slight deviation from complete fulfilment of all expectations (50%-90% compliance); a score of 1 indicated that only a minority of expectations were met (less than 50% compliance), while 0 points indicated that no expectations were met. The evaluation scale used in the evaluation was developed by the foil producer and described in the producer’s internal procedure. The maximal number of points that could be obtained was 39 (corresponding to 90–100% compliance). The third part of the questionnaire contained information about the supplier, such as category of supplied raw materials (raw materials directly and indirectly involved in production process), the location of the suppliers (country) and department in which the supplied materials were used in the foil producer’s plant [16]. The raw materials for the production, research & development (R&D) and logistics departments were taken into consideration. Before the main study, the evaluation questionnaire was validated among 10 suppliers.

Depending on the total points obtained, suppliers were divided into four categories: A (35–39 points–ca. 90–100% compliance), B (30–34 points–ca. 77–89% compliance), C (25–29 points–ca. 64–76% compliance), D (20–24 points–ca. 51–63% compliance). The evaluation results determined when the next supplier evaluation would take place, i.e. category A–next evaluation in one year, category B–next evaluation in six months, category C–next evaluation in three months, category D–further cooperation with the supplier denied. The time intervals for the next supplier evaluations were adopted according to the producer’s internal procedure and resulted from the risk assessment carried out in accordance with the requirements of the QMS [3].

### Suppliers characteristics

The study included 45 suppliers of raw materials for food packaging foil production. The suppliers who qualified for the evaluation had cooperated with the foil producer for at least two years, and before the evaluation they were audited at least once a year. New suppliers were excluded from the survey.

The predominant group (53.1%) was suppliers of raw materials directly involved in the foil production process, such as solvents, inks, low-density polyethylene (LDPE), high-density polyethylene (HDPE) and polypropylene (PP). The remaining 46.9% were suppliers of raw materials indirectly involved in production like acetone, stretch foil, proof paper, paper, pallets, mechanical parts, machines and devices. The suppliers were located in eight countries. Most of the suppliers were located in Germany (27.5%), and the fewest in Spain (5.0%). The locations of the other suppliers were as follows: the UK (22.5%), Italy (17.5%), the USA (9.4%), Greece (6.9%), France and Denmark (5.6% each). The supplied raw materials were used the most often in the departments of production (81.9%), than logistics (27.5%), and research and development (R&D) (8.6%).

### Statistical analyses

In the study a set of statistical tests was used, such as the Student’s t-test, PCA calculation, Spearman rank correlation coefficient, one-way analysis of variance ANOVA, Fisher’s least significant difference test (LSD), descriptive statistics, and Cronbach’s alpha. Such an approach was innovative and enabled in-depth analysis of the obtained results. The Student’s t-test was used to compare quantitative variables between the mean of economic and quality and processing criteria. Descriptive statistics methods, i.e., mean scores, median, mode and standard deviation (SD) were calculated for all assessments. To analyse the influence of the economic and quality and processing factors on the supplier evaluation results a PCA analysis (principal component analysis) was used. Additionally, to explain dependencies, the Spearman coefficient (rho) was calculated. The ANOVA and Fisher’s least significant difference test (LSD) were used to investigate the influence of the supplier’s characteristics on the evaluation result. The ANOVA calculations especially enabled to determine whether there were any statistically significant differences between the means of the evaluation results referring to supplier characteristics. The LSD test were used to find out exactly which groups of the suppliers are different from each other. Cronbach’s alpha was used to check reliability of each of the evaluation criteria, i.e., in our case economic and quality and processing criteria [40, 41]. All the calculations were done using Statistica 13.1 software. The significance was identified when p < 0.05.

## Results

The reliability coefficient Cronbach’s α for each of the evaluation criteria was calculated. In the case of economic criteria, Cronbach’s α = 0.825, in the case of quality and processing criteria, α = 0.862. Therefore, based on the calculation results both of the used criteria ware reliable.

The average scores were calculated for economic and quality and processing criteria. The mean score for criteria in the economic section was 2.32 (2.00–2.47; 66.7%-82% compliance), while for the quality and processing section it was 2.35 (1.86–2.55; 62%-85%) (Table 2). There were no statistical differences between the mean values of both sections (Student’s t-test, p = 0.266). None of the evaluated criteria received the highest average score of 3.0. However, the calculated maximum values showed that in each criterion some suppliers achieved the highest rating.

**Table 2:** Results of the descriptive statistics for the supplier criteria being evaluated.

No.	Supplier assessed criteria	Mean	% of compliance	Median	Mode	Min.	Max.	SD
**1**	**Economic criteria**							
1.1	Price	2.00	66.70%	2.00	2.00	1.00	3.00	0.74
1.2	Financial position	2.25	75.00%	2.00	2.00	1.00	3.00	0.61
1.3	Desire for business	2.46	82.00%	2.00	2.00	2.00	3.00	0.50
1.4	Procedural compliance	2.47	82.33%	2.00	2.00	2.00	3.00	0.50
1.5	Cooperation	2.33	77.66%	2.00	2.00	2.00	3.00	0.47
1.6	Business ethics	2.41	80.33%	2.00	3.00	1.00	3.00	0.63
**2**	**Quality and processing criteria**							
2.1	Material quality	2.54	84.66%	3.00	3.00	1.00	3.00	0.52
2.2	Lead time	1.86	62.00%	2.00	1.00	0.00	3.00	0.55
2.3	Certification	2.53	84.33%	3.00	2.00	1.00	3.00	1.24
2.4	Production facilities and capacity	2.40	80.00%	2.00	2.00	2.00	3.00	0.49
2.5	Technical capability	2.17	72.33%	2.00	2.00	1.00	3.00	0.56
2.6	Service/Technical assistance	2.44	81.33%	2.00	3.00	1.00	3.00	0.58
2.7	Warranties and claims policies	2.55	85.00%	3.00	3.00	2.00	3.00	0.50

Price (Table 2, no. 1.1, score 2.00, SD = 0.74, ca. 67% compliance) and financial position (Table 2, no. 1.2, score 2.25, SD = 0.61, ca. 75% compliance) were rated the lowest among economic criteria. The median value for these criteria was 2.00 and the maximum score was 3.00. None of them obtained a 0.00 score. The other criteria were rated at a similar level (2.25–2.47).

Among quality and processing criteria, the lowest score was given to delivery lead time (Table 2, no. 2.2, score 1.86, SD = 0.55, ca. 62% compliance), which was the lowest score among all evaluation criteria in the questionnaire. This was evidenced by both the lowest average (1.86) as well as the lowest minimal score (0.00). Among these criteria, technical capability also (Table 2, no. 2.5, received low scores (average 2.17, min. 1.00, ca. 72% compliance).

Among economic criteria, procedural compliance was the highest rated (Table 2. no. 1.4, score 2.47, ca. 82% compliance). Meanwhile the quality and processing criteria, warranties and claims policies (Table 2, no. 2.7, score 2.55, median 3.00, ca. 85% compliance) and raw material quality (no. 2.1, score 2.54, median 3.00, ca. 84% compliance) obtained the highest scores.

The total result showed that the category A of suppliers, i.e. those with the highest evaluation results and the highest % of compliance, accounted for 15% of evaluated suppliers. Category B corresponded to 37%, and C to 49% of suppliers. None of the suppliers was in the lowest D category.

**Fig 1:**
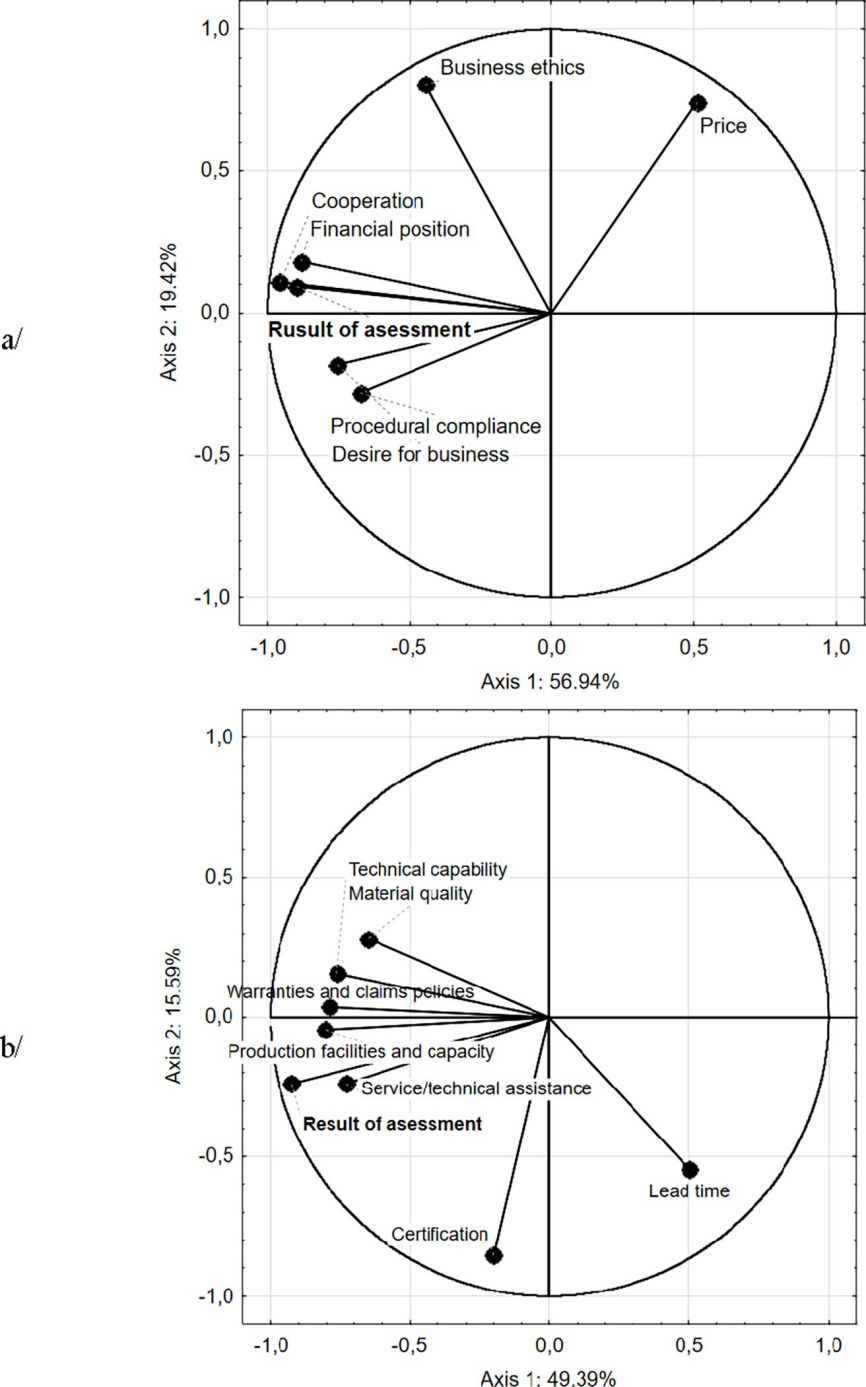
Influence of (a) economic, and (b) quality and processing criteria on the result of suppliers evaluation.

The study investigated the impact of particular criteria on the supplier evaluation results (PCA calculations). The PCA results showed correlations between the total result of the suppliers evaluation and economic factors (Fig 1A). For better description of the results, a high number of grouping variables was selected for the analysis. Axes 1 and 2 explained the correlation in 56.94% and 19.42%, respectively. Moreover, correlations obtained in PCA were additionally confirmed by Spearman’s correlation analysis. Among the economic factors, two factors had the strongest significant impact on the supplier evaluation result. They were cooperation (Spearman rank correlation coefficient rho = 0.816) and financial position (Spearman rank correlation coefficient rho = 0.730).

Other factors in the same half of the biplot, i.e. procedural compliance, desire for business and business ethics also had a significant, however slightly weaker, impact on the supplier evaluation results (Spearman rank correlation coefficient rho = 0.586, 0.547, 0.409, respectively). Only in the case of price, was a negative correlation found (Spearman rank correlation coefficient rho = –0.362). This indicated that the supplier evaluation score increased with the price of raw materials.

Fig 1B. indicated the main quality and processing factors that determined the results of the supplier evaluation. Axes 1 and 2 explained the correlations in 49.39% and 15.59%, respectively. Two factors: production facilities and technical capacity (Spearman rank correlation coefficient rho = 0.713), and warranties and claims policies (Spearman rank correlation coefficient rho = 0.685) had the most significant impact on the results. Other factors in this half biplot i.e. service/technical assistance (Spearman rank correlation coefficient rho = 0.648), technical capability (Spearman rank correlation coefficient rho = 0.592), material quality (Spearman rank correlation coefficient rho = 0.520) and certifications (Spearman rank correlation coefficient rho = 0.485) also showed a positive, however slightly weaker, impact on evaluation results. Only in the case of lead time, was a negative correlation found (Spearman rank correlation coefficient rho = -0.416), i.e. with the extension of order lead time (the score in the questionnaire was 1) supplier evaluation results increased.

The impact of the supplier category on the evaluation results was analysed depending on the type of raw materials supplied (no. 3.1, i.e. raw materials directly and indirectly involved in production), the location of the suppliers (no. 3.2) and the department when supplied raw materials were used (no. 3.3, i.e. production, R&D, logistics).

**Fig 2:**
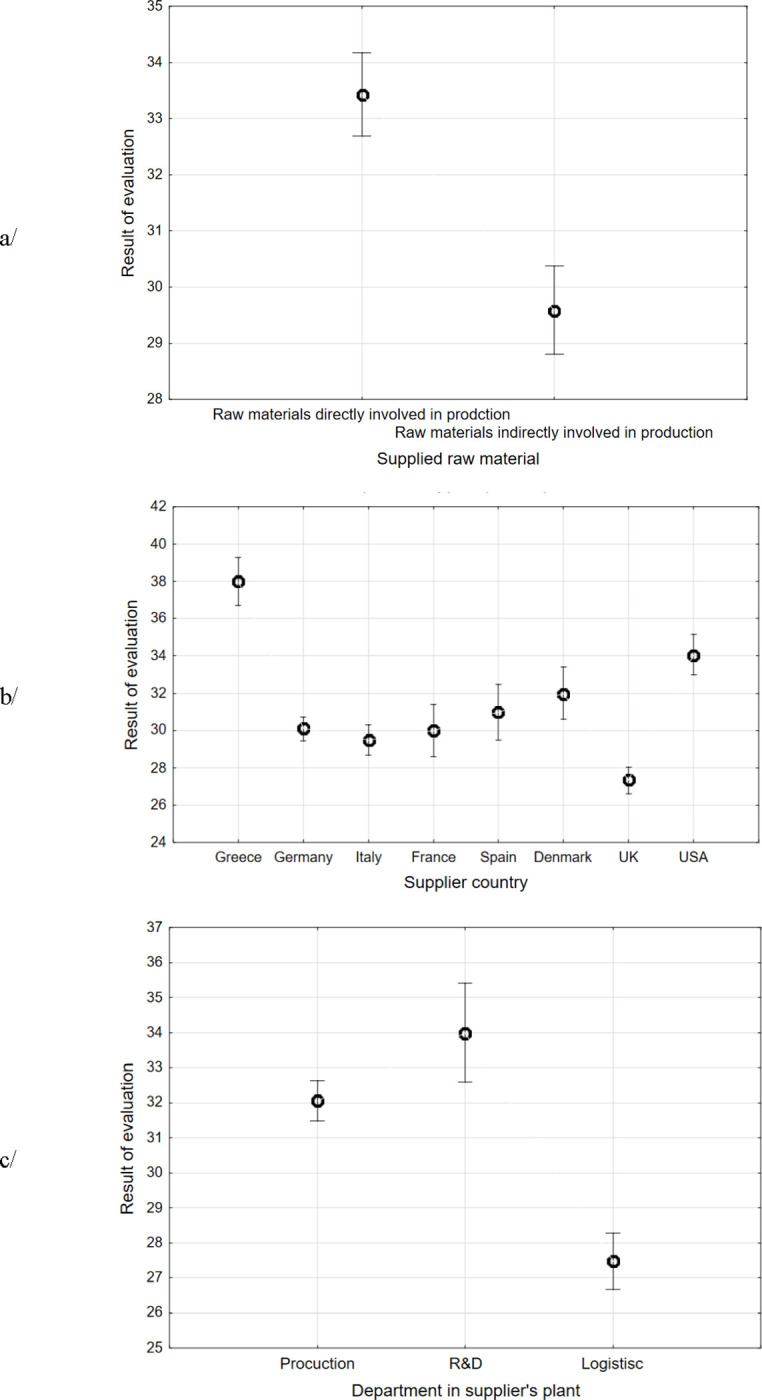
Influence of the information about suppliers on evaluation results: a) type of raw materials supplied, b) supplier location, c) department where the raw materials were used.

All the supplier characteristics i.e. supplied materials (3.1), the location of the supplier (3.2) and the departments when supplied materials were used (3.3) significantly influenced the total results of the supplier evaluation (p value ANOVA: supplied materials, p = 0.000; location, p = 0.000; department, p = 0.0000). The Fisher’s least significant difference test (LSD) was then used to determine the differences between supplier subcategories. In the case of the supplied materials category (no. 3.1) (Fig 2A), the suppliers of raw materials directly involved in production achieved the highest scores reaching 33.43 points (SD = 0.33) and suppliers of raw materials indirectly involved in production achieved 29.58 (SD = 0.35). Suppliers of materials directly and indirectly involved in production were classified in category B, and their next evaluation should take place in the next six months. The calculations showed that the ratings of all categories of suppliers differed significantly (LSD p = 0.000).

In the case of the supplier location (no. 3.2) (Fig 2B), the highest average score was obtained by suppliers from Greece (38.00, SD = 0.64), then from the USA (34.06, SD = 0.55), Denmark (32.00, SD = 0.71), Spain (31.00, SD = 0.75), Germany (30.09, SD = 0.32), France (30.00, SD = 0.71), Italy (29.50, SD = 0.40), and the UK (27.33, SD = 0.35). The calculations indicated that the results of the evaluation of suppliers from Greece and the USA were significantly higher than all other suppliers (Fisher’s LSD test for Greece as refers to all suppliers, LSD p = 0.000, for USA as refers to Greece, Germany and Italy, LSD p = 0.000, to Denmark, LSD p = 0.023 and Spain, LSD p = 0.001). On the other hand, the results of the UK suppliers were the lowest and differed significantly from the results of the other suppliers (Fisher’s LSD test for UK as refers to all suppliers LSD p = 0.000). The results of suppliers from Germany, Italy, France and Spain did not differ significantly from each other (LSD p = 0.255; LSD p = 0.907; LSD p = 0.271, respectively).

In the case of the department category, i.e. where raw materials were used (no. 3.3) (Fig 2C), the highest average score 34.00 points (SD = 0.67) was obtained by the raw materials suppliers for the R&D departments. The calculations showed that this assessment differed from suppliers to other departments such as production (LSD p = 0.008) and logistics (LSDp = 0.000). The second was an assessment of raw materials suppliers for production departments. The mean score was 32.04 points (SD = 0.27) and it differed significantly from the assessments of suppliers to other departments (for R&D, LSD p = 0.008 and logistics LSD p = 0.000). The lowest mean score 27.50 points was obtained by the logistics suppliers.

## Discussion

Many advanced methods of suppliers evaluation are described in the literature. These are: Analytic hierarchy process (AHP), analytic network process (ANP), data envelopment analysis (DEA), total cost of ownership (TCO) and artificial neural network (ANN) [18, 22, 23, 42, 43]. Simple methods include multi-criteria decision-making methods (MCDM), i.e. point and point-weight methods [33, 34, 37]. Many of them are used in practice [10, 34, 35, 44–47]. There is no one method that is considered to be clearly better than another, although some techniques are better suited to certain decision problems. Nevertheless, the Covid-19 pandemic has made existing control methods often impossible to use. Therefore, methods and tools of remote evaluation, might be more useful than others during pandemic time.

In the presented procedure of remote evaluation, economic, quality and processing criteria were taken under consideration. The calculations showed a similar average level of fulfilment of both criteria, while the fulfilment of individual subcriteria was varied. In economic criteria, price was the lowest rated subcriterion. Purchasing price is usually considered as a major determinant of a company’s ability to achieve competitiveness and high profit margins [24]. Price as an important element of supplier evaluation and selection was emphasised by many authors [19, 42, 43, 47–50]. However, in this study, the PCA and Spearman’s calculations indicated that price as only one economic criterion did not affect the supplier evaluation score. This might be related to the acceptance of the agreed prices at the stage of qualifying suppliers for cooperation [44]. Nevertheless, the criteria for cooperation and financial position were of great importance in the PCA calculations. The significant importance of these criteria was also indicated by Hashemian et al. [43] in studies on the evaluation of milk suppliers and in other studies [51]. This was probably related to the suppliers stable position on the market, supply chain continuity, the ability to cope with critical financial situations, financial liquidity and the quality of cooperation and relations with the supplier [42].

The high level of the producer’s relationship with its suppliers was also evidenced by business ethics. The business ethics criterion was emphasised in other studies as well [26]. Business ethics covers many aspects: applying ethical standards such as a code of ethics, legal and moral principles, respect for confidentially of supplier information, respect for partners, justice, politeness of staff, transparency in activities, clear communication, and fair but firm negotiations, human rights, labour standards and environmental responsibility [52, 53]. The fact that the foil producer paid attention to the suppliers business ethics aspects deserves a positive emphasis, especially as these issues have not always been taken into account by other food sector operators [54, 55]. Similarly, positive evaluations and a significant impact on the result of the supplier evaluation were shown in the cases of procedural compliance and desire for business. Positive results in these criteria indicated successful supplier selection prior to cooperation.

Almost all quality and processing criteria, except for lead time, influenced the evaluation of suppliers. Several subcriteria were very important. These were subcriteria covering supplier technical aspects such as production facilities and capacity, warranties and claim policies, service/technical assistance and technical capability. The importance of the criteria for production facilities and capacity and for technical capacity were also emphasised by other authors [50, 56]. This was mainly related to production efficiency, as a large producer preferred to cooperate with suppliers who could fulfil current and future orders. The importance of results of these criteria evaluation in other studies were very diverse, from highly rated in Hashemian et al. [43] study, to insignificant in Galinska & Pisarek-Bartoszewska study [56]. The same applies for criteria of service/technical assistance. In Acar et al. study [24], this criterion was an important factor, but in Galinska & Pisarek-Bartoszewska study [56] it did not matter. The subcriterion of warranties and claim policies in the evaluation of suppliers in textile industry plants was not considered at all [24]. In the case of the food packaging foil producer, this criterion strongly influenced the result of the supplier evaluation and received the highest rating. Considering the different results and the use of these criteria in different manufacturers, it should be emphasised that the technical criteria should be used in the supplier evaluation depending on the current needs and specifics of the supplied raw materials.

The quality subcriterion was one of the most frequently used to evaluate suppliers and deliveries [12, 31, 35], particularly in the food industry [25]. Quality is often connected with having certifications ensuring that the requirements have been met. The results of the quality assessment and supplier certifications were not always positive [47, 57]. However, a study from Tanzania showed that in some regions poor-quality materials were rarely mentioned as a barrier to supplier relationship management [32]. The PCA and Spearman’s calculations of this study’s results showed the large impact of certification on supplier evaluation rating. In other studies it was shown that certification positively affected the implementation of processes, better organisational performance and employee training [58, 59]. Only a few studies indicated no significant differences between the evaluation of certified and non-certified enterprises [4].

Among the evaluated suppliers, the lead time criterion was rated the lowest. However, the PCA and Spearman’s calculations indicated that this subcriterion had no effects on supplier evaluation. Some authors indicated that lead time was not an important factor in supplier evaluation [28, 59], yet others pointed out the opposite [25, 51, 57]. Differences might have resulted from the type of raw materials supplied. In the case of a food packaging foil producer, the delivered raw materials had a long shelf life. On the other hand, in the case of fresh food raw materials delivery time was a critical criterion [33, 37].

It was also shown that the relationship between the location of the suppliers, the type of raw materials supplied and the target department for the use of raw materials in the foil producer’s plant. Geographical distance between supply chain partners is an important factor in the organisation of supply particularly in food industry enterprises [25, 44, 60], but also in other industries [56]. However, this was not important for the food packaging foil producer. The calculations indicated that long distance and transport time did not lower the supplier rating. Domestic suppliers, i.e. in Greece, received positive evaluations, as well as suppliers from the farthest country, i.e. the USA. The lack of influence of location on the supplier rating was probably related to the lack of stringent transport requirements in this case because e.g. components for production of packaging foil do not need to be stored at any specific temperature or short time frame. The long distance from the supplier might only make it difficult to implement the supplier audit programme and therefore the use of the remote evaluation method might be very useful.

Suppliers of raw materials for the R&D department obtained the best evaluation scores, which was different from other destinations. These results highlighted the importance of the R&D department of the foil producer in conducting its own research [28, 61]. The necessity of having an R&D department is aimed at conducting own research in the fields of control and improvement of current products and the development of new ones [3]. It could be not without significance that the foil producer operates in a country, where the economic development level, measured by GDP per capita, was below average while the share of actively innovation-driven companies was above average [61, 62].

Post hoc calculations showed that the suppliers of raw materials directly and indirectly related to production obtained the best scores. This proved the foil producer’s diligence in meeting the requirements of the QMS, i.e. monitoring raw materials [3] and minimising the risk associated with defective raw materials [16]. The indication of the supplier’s status (A, B, C, D), corresponding to the level of compliance, was related to the frequency of supplier evaluation.

There are no reports presenting methods of evaluation of raw materials suppliers for producers of food packaging foil. There are also scant reports describing methods of evaluation in special conditions when regular audits are limited or cannot to be carried out at all, as during the Covid-19 pandemic. This study fills this gap, presenting a method of remote evaluation. The obtained results will be useful for auditors of producers of food packaging materials, auditors conducting certification audits, quality managers and representatives of official food control in special conditions when regular audits are limited or cannot be carried out as during the Covid-19 pandemic or others to come in the future. This is a real and practical advantage of developed procedure. Nevertheless, this study also has some limitations. The evaluated suppliers were only located in the EU, the UK and the USA. Suppliers from other areas, e.g. from Asia or South America, may have different production conditions and involve a different business policy. The surveyed foil producer was a large enterprise, and the operating conditions of smaller ones might be different in their approach to the implementation of the QMS and to meeting the expectations of their recipients in the context of product quality. Finally, the presented supplier evaluation criteria may need to be modified in the case of other food industry sectors.

## Conclusions

The presented method of supplier evaluation was shown to be a useful procedure to fulfil the requirements of the QMS in terms of supplier supervision during the Covid-19 pandemic. Among the economic criteria, procedural compliance was rated the highest and the lowest rated was price of raw materials, while among the quality and production criteria, the highest average score was given to the warranties and claims policies, and the lowest one to the lead time. The results of the study showed that the quality of the raw materials directly used in the production of food packaging foil was adequate. Therefore, their use ensure production of packaging foil and finally packaged meat products of adequate quality and safety.

Not all analysed criteria had the same impact on the final supplier rating. The strongest influence on the supplier evaluation results had criteria of cooperation and financial position, as well as production facilities and capacity, warranties and claims policies. The applied remote evaluation method showed that the suppliers of raw materials met the requirements at various levels. The highest ratings obtained suppliers of raw materials directly involved in production–suppliers from Greece, the USA and Denmark–as well as suppliers of raw material for the R&D department. This indicated the need for systematic evaluation of suppliers in every part of the food supply chain to ensure the adequate quality and safety of food products.

The presented procedure occurred to be useful for remote evaluation of quality, processing and economic criteria in qualification of suppliers during the Covid-19 pandemic. It may inspire other producers of food packaging materials to continuing supervision over of their suppliers while regular methods of control are limited. Final conclusions of the research can be highlighted in bullets: 1/ The questionnaire enables remote evaluation of most important criteria in suppliers qualification; 2/ The highest ratings obtained suppliers of raw materials directly involved in production; 3/ Systematic evaluation of suppliers is possible while regular methods of control are limited; 4/ Suppliers distant location did not disturb their evaluation when remote procedure is used.
